# A rare case of primary hydatid cyst of the adrenal gland: Case report and review of the literature

**DOI:** 10.1016/j.ijscr.2025.111669

**Published:** 2025-07-11

**Authors:** Iraj Feizi, Atabak Sedigh-Namin, Ghazaal Ghasemi, Mirsalim Seyedsadeghi, Sonia Sharifi Namin, Alireza Bagheri Toularoud

**Affiliations:** a Department of Surgery, School of Medicine, Ardabil University of Medical Sciences, Ardabil, Iran; b Students Research Committee, School of Medicine, Ardabil University of Medical Sciences, Ardabil, Iran; c General Practitioner, Faculty of Medicine, Islamic Azad University, Ardabil Branch, Ardabil, Iran; d Department of Anatomical Sciences and Pathology, School of Medicine, Imam Reza Hospital, Ardabil University of Medical Sciences, Ardabil, Iran

**Keywords:** Hydatid cyst, Echinococcus granulosus, Adrenal gland, Case report

## Abstract

**Introduction and importance:**

Hydatid disease, caused by Echinococcus granulosus, is endemic in several regions worldwide, including the Middle East. While the liver and lungs are commonly affected, adrenal gland involvement is extremely rare and often misdiagnosed.

**Case presentation:**

We report a 22-year-old male from a rural area who presented with paraesthesia in his feet. Imaging revealed a large cystic lesion near the right adrenal gland. Further diagnostic workup, including CT scan and serology, suggested a chronic hydatid cyst. The patient received preoperative albendazole followed by successful surgical excision. Histopathological findings confirmed the diagnosis.

**Clinical discussion:**

Adrenal hydatid cysts are rare, accounting for less than 1 % of all hydatid disease cases. Clinical presentation is usually non-specific, making imaging essential for diagnosis. This case underlines the importance of considering hydatid cysts in the differential diagnosis of adrenal masses, especially in endemic regions.

**Conclusion:**

Early diagnosis and proper management of adrenal hydatid cysts can prevent serious complications. A multidisciplinary approach and awareness of atypical presentations are crucial for effective treatment.

## Highlights

• Adrenal hydatid cysts are extremely rare, comprising less than 1% of hydatid disease cases.
• This case emphasizes the need to consider parasitic etiologies in the differential diagnosis of adrenal masses in endemic areas.
• Imaging, particularly contrast-enhanced CT, played a pivotal role in preoperative diagnosis.
• The combination of albendazole therapy and complete surgical excision led to a favorable outcome without recurrence.

## 1 Introduction

This case report has been reported in accordance with the SCARE 2025 criteria to ensure a structured and comprehensive presentation [1]. The hydatid cyst is a recognized zoonosis native to some regions, including North Africa, the Middle East, Central Asia, Australia, and Latin America [2,3]. Hydatid cysts result from the larval stage of Echinococcus granulosus. They possess the capacity to differentiate into any organ or tissue inside the body; yet, in humans, the liver and lungs are the organs most frequently affected. Even in our region, where echinococcosis is endemic, adrenal involvement is exceptionally rare [2]. The consideration of adrenal hydatid cysts might facilitate the prompt diagnosis and treatment of the patient, given the existence of differential diagnoses including pheochromocytoma, adrenal adenoma, adrenal myelolipoma, and tuberculous adrenalitis [3]. In our presentation, we describe the case of a 22-year-old male with a growing mass in the adrenal. This report highlights the clinical and radiological characteristics of the case, details the surgical intervention, and provides a brief review of the relevant literature.

## 2 Case report

A 22-year-old male, from a rural area, with no notable past medical history, presented to the clinic with complaints of paraesthesia in both of his feet. He described the sensation as a tingling and numbness, which had gradually progressed over the past three weeks. He had no history of fever, weight loss, or abdominal pain. On physical examination, no signs of acute distress or abnormal findings were noted. However, neurological examination revealed decreased sensation in the feet, indicating possible nerve involvement.

Given the patient's rural background, the physician considered various potential causes, including vitamin deficiencies, metabolic disorders, and parasitic infections. A comprehensive laboratory workup was requested, including a complete blood count (CBC) with differential, liver function tests (LFTs), and a lipid profile. The patient's lipid profile showed markedly elevated triglyceride levels. Further investigation into liver function revealed no significant abnormalities; however, the elevated triglycerides raised concern for possible hepatic pathology.

To evaluate the abdominal region further, an abdominal ultrasound was performed. The ultrasound revealed a grade 2 fatty liver and a focal heterogeneous lesion in the inferior portion of the sixth lobe of the liver, adjacent to the right adrenal gland, measuring approximately 81 × 78 mm. The differential diagnosis at this point included adrenal adenoma, pheochromocytoma, and, given the patient's rural living environment, a hydatid cyst.

**Fig. 1:**
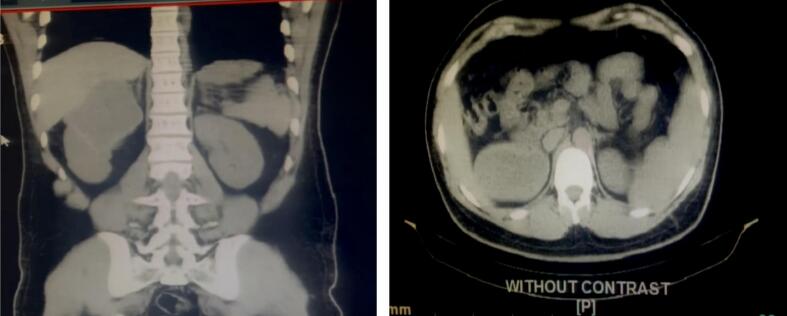
Abdominal CT scan demonstrating a heterogeneous cystic lesion with internal septations and echogenic foci, consistent with a stage 4 chronic hydatid cyst.

In light of the ultrasound findings, the patient underwent additional imaging studies. A contrast-enhanced abdominal CT scan was performed, revealing a heterogeneous cyst with a stratified internal structure and echogenic foci, characteristic of a stage 4 chronic hydatid cyst (Fig. 1). The cyst measured 85 × 83 × 82 mm, with a volume of approximately 300 cc, and was located between the liver and right kidney, possibly originating from the mesenteric region. This imaging result strongly suggested a hydatid cyst, leading to the exclusion of adrenal adenoma and pheochromocytoma.

Further laboratory tests, including VMA (Vanillylmandelic acid), metanephrine, and IgG for echinococcosis, were conducted. The VMA and metanephrine levels were negative, effectively ruling out pheochromocytoma. The IgG test for echinococcosis also returned negative, but considering the clinical context and the endemic nature of hydatid disease in the region, a treatment plan targeting hydatid cysts was initiated. The patient was started on oral albendazole for one month to reduce the size of the cyst and prevent complications.

After one month of medical therapy, the patient was admitted to the hospital for surgical intervention. Throughout his hospitalization, his vital signs remained stable (blood pressure = 100/60 mmHg, heart rate = 83 bpm, respiratory rate = 16 breaths per minute). He did not experience any flushing, palpitations, diaphoresis, or hypertensive crises, which further supported the exclusion of pheochromocytoma.

**Fig. 2:**
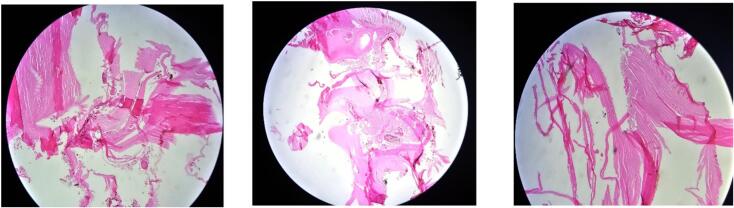
Degenerated acinoccal cysts that contain acellular laminated membrane, debris and protoscolices fragments.

On the second day of hospitalization, the patient underwent surgical resection of the cyst. A laparotomy was performed, and the hydatid cyst located on the right adrenal gland was successfully excised. The cyst appeared intact, with no evidence of rupture or leakage during the procedure. The excised specimen was sent for histological examination. The pathology report confirmed the diagnosis of a hydatid cyst, with typical findings including laminated layers and hydatid sand within the cyst (Fig. 2).

After the surgery, the patient was transferred to the ICU for monitoring and was later moved to the main ward on the following day. His postoperative course was uneventful, and he was discharged from the hospital after three days in good condition. The patient was prescribed oral albendazole for an additional three months to prevent recurrence.

Follow-up visits were scheduled at one-month intervals. During these visits, the patient remained asymptomatic, and there were no signs of cyst recurrence or complications. His neurological symptoms improved gradually, and he reported no further tingling or numbness in his feet.

## 3 Discussion

Hydatid cysts, primarily caused by Echinococcus granulosus, are a global health concern with a variety of clinical manifestations depending on the organ affected [4]. While the liver and lungs are the most commonly involved organs, the adrenal glands are an extremely rare location for hydatid cysts, accounting for less than 1 % of cases [5]. These cysts are typically asymptomatic, often discovered incidentally during imaging studies conducted for other conditions. When symptoms do occur, they are usually due to pressure effects or complications such as rupture, which can lead to severe consequences, including anaphylaxis and bleeding [6].

The challenge in diagnosing adrenal hydatid cysts lies in their often subtle clinical presentation and the difficulty in distinguishing them from other cystic lesions. Imaging techniques such as ultrasonography, CT, and MRI play crucial roles in the diagnostic process [7]. Ultrasonography remains the first-line imaging modality with a high sensitivity of 93–98 %, providing valuable information regarding cyst characteristics such as the “daughter cysts” and “water lily sign”. The World Health Organization (WHO) classification, which stages hydatid cysts into six categories (CE1, CE2, CE3a, CE3b, CE4, and CE5), is essential for determining the cyst's activity level and planning treatment strategies [8]. This classification helps distinguish between active cysts, which may respond well to treatment, and inactive cysts, which are typically non-viable. We have summarized these stages in Table 1, providing clarity on their clinical implications.

**Table 1:** Summary of the World Health Organization (WHO) classification of hydatid cysts into six stages (CE1 to CE5), indicating cyst activity status and their clinical implications for treatment planning.

Stage (CE)	Characteristics	Cyst type	Response to treatment
CE1	Unilocular cysts	Unilocular cysts	Effective treatment with albendazole
CE2	Multivesicular cysts with daughter cysts	Multivesicular cysts	Effective treatment with albendazole
CE3a	Cysts beginning to degenerate with water lily sign	Transitional cysts	Effective treatment with albendazole
CE3b	Predominantly solid cysts with daughter cysts	Transitional cysts	Limited treatment, requires cystectomy
CE4	Cysts with signs of solidification and increasing calcification	Solidified cysts	Ineffective treatment with albendazole
CE5	Completely calcified cysts with involuted content	Calcified cysts	Ineffective treatment

Additionally, a detailed review of cases of adrenal hydatid cysts from 1985 to 2025 is presented in Table 2, illustrating the rarity of this condition and offering valuable insights into its epidemiology, treatment approaches, and outcomes. This table highlights key findings, treatment modalities, and complications reported in previous studies, which can serve as a guide for future management of similar cases [9].

**Table 2:** Review of cases of adrenal hydatid cysts from 1985 to 2025.

Author	Age	Sex	Environment	Contact with animals	Symptoms	Hypereosinophilia	Serology	Side	Size (cms)	Primary/Secondary	Treatment by albendazole	Surgical access	Follow up (months)	Recurrence
Fitzgerald EJ (1985)	48	M	N/A	N/A	Right hypochondruim pain	N/A	Positive	Right	18	Primary	N/A	Open surgery	N/A	N/A
Sroujieh AS (1990)	50	N/A	N/A	N/A	N/A	N/A	N/A	N/A	N/A	Secondary	N/A	Open surgery	N/A	N/A
Schoretsanitis G (1998)	48	M	Rural	Yes	Right hypochondruim pain	No	Negative	Right	9.5	Primary	No	Open surgery	N/A	N/A
Defechereux T (2000)	37	F	N/A	N/A	Left Flank Pain	No	N/A	Left	5	Secondary	No	Laparoscopy	N/A	N/A
C.Ö. Yeniyol (2000)	51	F	N/A	N/A	Left Flank pain	N/A	Positive	Left	6	Primary	N/A	N/A	N/A	N/A
el Idrissi Dafali A (2002)	28	N/A	Urban	No	Right hypochondruim pain	Yes	Positive	Right	5,7	Primary	No	Open surgery	48	No
Escudero MD (2002)	40	F	Urban	No	Hypertension	N/A	N/A	Left	10	Primary	N/A	Open surgery	12	No
	53	F	N/A	N/A	Right hypochindrium pain	N/A	Positive	Right	8	Primary	N/A	N/A	16	No
	80	M	N/A	N/A	Right hypochondrium pain	N/A	Positive	Right	10	Secondary	N/A	N/A	16	No
Mufide Nuran Akcay (2003)	48	F	N/A	N/A	Incidental	N/A	Negative	Right	4,5	Secondary	N/A	N/A	16	No
	61	F	N/A	N/A	Left hypochondruim pain	N/A	Positive	Left	6,5	Primary	N/A	N/A	16	No
	18	F	N/A	N/A	Incidental	N/A	Negative	Right	10	Secondary	N/A	N/A	16	No
	15	M	N/A	N/A	Right flank pain	N/A	Positive	Right	12	Primary	N/A	N/A	16	No
	18	M	N/A	N/A	Left flank pain	N/A	Positive	Left	20	Primary	N/A	N/A	16	No
	41	M	N/A	N/A	Right hypochondruim pain	N/A	Positive	Right	5	Secondary	Yes	N/A	16	No
	28	F	N/A	N/A	Incidental	N/A	Negative	Right	20	Primary	N/A	N/A	16	No
Gürdal M (2004)	48	F	N/A	N/A	Right flank pain	No	Negative	Right	6	Primary	N/A	Open surgery	12	No
Recai Gurbuz (2005)	47	F	N/A	N/A	Left flank pain	N/A	N/A	Left	7,8	Primary	N/A	Open surgery	Yes	N/A
H. Bedioui (2005) Ali Horchani (2006)	20	M	N/A	N/A	Epigastralgia	No	Positive	Right	5,6	Primary	N/A	Open surgery	24	No
	50	M	N/A	N/A	Left hypochondruim pain	No	Negative	Left	6	Primary	N/A	Open surgery	36	No
	24	M	N/A	N/A	Right flank pain	N/A	Positive	Right	5,6	Primary	N/A	Open surgery	24	No
	47	M	N/A	N/A	Left flank pain	N/A	Negative	Left	8	Primary	N/A	Laparoscopy	24	No
	55	M	N/A	N/A	Right flank pain + hypertension	N/A	Negative	Right	6	Primary	N/A	Open surgery	24	No
	59	M	N/A	N/A	Left flank pain	N/A	Positive	Left	7	Secondary	N/A	Open surgery	24	No
	54	M	N/A	N/A	Left flank pain	N/A	Negative	Left	6	Primary	N/A	Open surgery	24	No
	44	F	N/A	N/A	Right hypochondruim pain	N/A	Negative	Right	5	Primary	N/A	Open surgery	24	No
Nikica Grubor (2006)	52	M	N/A	N/A	Epigastralgia	No	N/A	Right	4,4	Primary	N/A	Open surgery	N/A	N/A
Ozarmagan S (2006)	54	F	N/A	N/A	Hypertension	N/A	Positive	Right	5	Primary	Yes	Open surgery	N/A	N/A
Safioleas (2006)	61	F	N/A	N/A	Epigastralgia/Hypertension	Yes	Positive	Left	5,8	Primary	Yes	Open surgery	6	No
Tsaroucha AK (2007)	56	M	Rural	Yes	Hypertension	No	Negative	Left	7	Primary	No	Open surgery	12	No
Shintaro Maru (2007)	79	F	Urban	N/A	Impaired general condition	No	Positive	Right	5,5	Primary	No	Open surgery	N/A	N/A
Gianlorenzo Dionigi (2007)	68	F	N/A	Yes	Left Flank pain	No	Positive	Left	3	Primary	Yes	Laparoscopy	6	N/A
Ruiz-Rabelo JF (2008)	70	F	N/A	N/A	Left hypochondruim pain + Fever	No	Negative	Left	9	Secondary	No	Open surgery	N/A	No
Tamotsu Kamishima (2009)	77	M	N/A	N/A	Hypertension	No	N/A	Right	6	Primary	No	Laparoscopy	N/A	N/A
O. Baraket (2010)	38	F	Rural	Yes	Left Flank pain	N/A	N/A	Left	7	Primary	N/A	Open surgery	36	No
B Geramizadeh (2011)	49	F	N/A	N/A	Left Flank pain + hypertension	No	N/A	Left	8,2	Primary	No	N/A	2	No
Limaiem F (2012)	55	F	N/A	N/A	Left hypochondruim pain	No	N/A	N/A	12	Secondary	N/A	N/A	N/A	N/A
Fadl Tazi (2012)	64	M	N/A	N/A	Left flank pain + hypertension	Yes	Negative	Left	14,5	Primary	Yes	Open surgery	24	No
Maral Mokhtari (2012)	66	F	N/A	N/A	Right flank pain + hypertension	No	N/A	Right	5	Primary	No	Open surgery	N/A	N/A
Huang M (2013) Abdulla Darwish [11]	45	M	N/A	N/A	Incidental	N/A	N/A	Right	9,5	Primary	No	Open surgery	24	N/A
	56	F	N/A	N/A	Right hypochondruim pain	N/A	N/A	Right	11,2	Primary	No	Open surgery	24	N/A
	30	F	N/A	N/A	Hyperemesis gravidarum	No	Negative	Right	12	Primary	No	Open surgery	N/A	N/A
Babinska A (2014)	47	F	N/A	N/A	Hypertension	Yes	N/A	Right	6,8	Primary	N/A	Laparoscopic	132	No
Santosh Kumar (2014)	51	F	N/A	Yes	Left hypochondruim pain	No	Positive	Left	N/A	Primary	Yes	Laparoscopic	6	No
Afshin Mohammadi (2014)	N/A	M	N/A	N/A	Hypertension	N/A	N/A	Left	13	Primary	Yes	Open Surgery	6	No
Walter Nardi (2015)	55	M	N/A	N/A	Back pain	No	Negative	Left	6,5	Primary	N/A	Laparoscopic	N/A	N/A
Ammar Mahmoudi (2015)	76	F	Rural	N/A	Right hypochondruim pain	N/A	N/A	Right	5	Secondary	No	Open surgery	24	No
Silke Spahn (2016)	78	M	N/A	N/A	Incidental	No	Positive	Right	7,4	Primary	Yes	Open surgery	24	No
Gaurav Prakash (2016)	35	M	Rural	Yes	Right flank pain	No	N/A	Right	16	Primary	Yes	Open surgery	N/A	N/A
Fatehi Elnour Elzein [8]	44	M	N/A	Yes	Right flank pain	N/A	Negative	Right	10	Primary	Yes	Open surgery	N/A	N/A
Sami Akbulut [9]	64	M	N/A	N/A	Vague abdominal pain	No	Positive	Right	15	Primary	Yes	Open surgery	24	N/A
Giovanni Aprea (2016)	78	F	Urban	No	Right flank pain	No	N/A	Right	3,4	Primary	Yes	Laparoscopic	N/A	N/A
Ann-Katrin Seidel (2017)	16	M	N/A	N/A	Incidental	No	Positive	Right	7	Primary	Yes	Open surgery	N/A	N/A
Sami Akbulut [7]	64	M	Urban	No	Right hypochondruim pain	N/A	Positive	Right	7	Primary	Yes	Open surgery	12	No
Skander Zouari [6]	55	M	Urban	No	Left hypochondruim pain	No	Negative	Left	12	Primary	No	Open surgery	12	No
Houcine Bouchaala [5]	48	M	N/A	No	Right sided abdominal pain	N/A	N/A	Right	10.6	primary	Yes	Open surgery	N/A	No
Abasin Tajmalzai [4]	18	M	N/A	Yes	Right upper quadrant pain	N/A	Positive	Right	6	Primary	Yes	Open surgery	N/A	No
Present case (2025)	22	M	Rural	Yes	Paraesthesia	No	Negative	Right	8.5	Primary	Yes	Open surgery	3	No

Although traditionally open surgery (laparotomy) has been favored due to concerns of cyst rupture and dissemination, several case reports since the early 2000s have demonstrated the successful use of laparoscopic approaches in selected cases. Laparoscopic resection has been performed safely for adrenal hydatid cysts, especially when the cyst is small, well-encapsulated, and without extensive adhesion to surrounding structures.

Regarding treatment, surgical intervention remains the cornerstone for managing adrenal hydatid cysts. Laparoscopic resection and laparotomy are both viable options, with the choice depending on cyst size, location, and the presence of complications. The goal is complete cystectomy while preserving the adrenal gland, as removal of the gland is typically only necessary in complicated or non-resectable cases. In some instances, preoperative injection of hypertonic saline into the cyst may help inactivating the scolices and daughter cysts, reducing the risk of intraoperative spillage and enhancing the success of cyst removal [10].

In terms of medical treatment, albendazole has shown effectiveness in treating hydatid cysts, particularly those in the active stages (CE1, CE2, and CE3a), although its efficacy is lower in cysts that are predominantly solid (CE3b) or calcified (CE4, CE5). The role of albendazole should be carefully considered in the context of the patient's overall clinical presentation and cyst characteristics.

The management of hydatid disease requires a multidisciplinary approach, involving surgeons, radiologists, and infectious disease specialists. Public health measures, including the treatment of infected animals and vaccination programs, are essential for controlling the spread of this parasitic disease [12].

## 4 Conclusion

Adrenal hydatid cysts remain a rare but important consideration in the differential diagnosis of adrenal masses. Although often asymptomatic, these cysts can cause significant morbidity due to their potential complications. Early diagnosis through appropriate imaging techniques and classification, combined with timely surgical intervention, remains the most effective approach to managing these challenging cases.

## Author contribution

Atabak Sedigh-namin: Data collection, data analysis, and manuscript writing, and first author.

Alireza Bagheri Toularoud: Study design, data interpretation, and manuscript review, and corresponding author.

Iraj feizi: Study concept and design, data interpretation, and manuscript review, and first author.

Ghazaal Ghasemi: Data interpretation.

Mirsalim Seyedsadeghi: Data interpretation.

Sonia Sharifi Namin: Data interpretation.

## Consent

Written informed consent was obtained from the patient for publication of this case report and accompanying images. A copy of the written consent is available for review by the Editor-in-Chief of this journal on request.

## Ethical approval

This study was conducted in accordance with the ethical principles outlined in the Research Ethics Committees of Ardabil University of Medical Sciences. As this research, it was determined to be exempt from formal ethical review by the Research Ethics Committees of Ardabil University of Medical Sciences.

## Guarantor

Alireza Bagheri Toularoud.

## Research registration number

None.

## Funding

None.

## Conflict of interest statement

Authors have no conflict of interest to declare.
